# Supplementary material for: Genome-wide association study of cardiac troponin I in the general population
Source: Hum Mol Genet. 2021 May 7;30(21):2027–39. doi: 10.1093/hmg/ddab124 (PMC8522636; doi:10.1093/hmg/ddab124)
Supplement: HMG-2021-AM-00007_MOKSNES_SUPPLEMENTAL_ddab124 [file hmg-2021-am-00007_moksnes_supplemental_ddab124.doc]

# Supplementary Material

Figures S1, S2

Author contributions


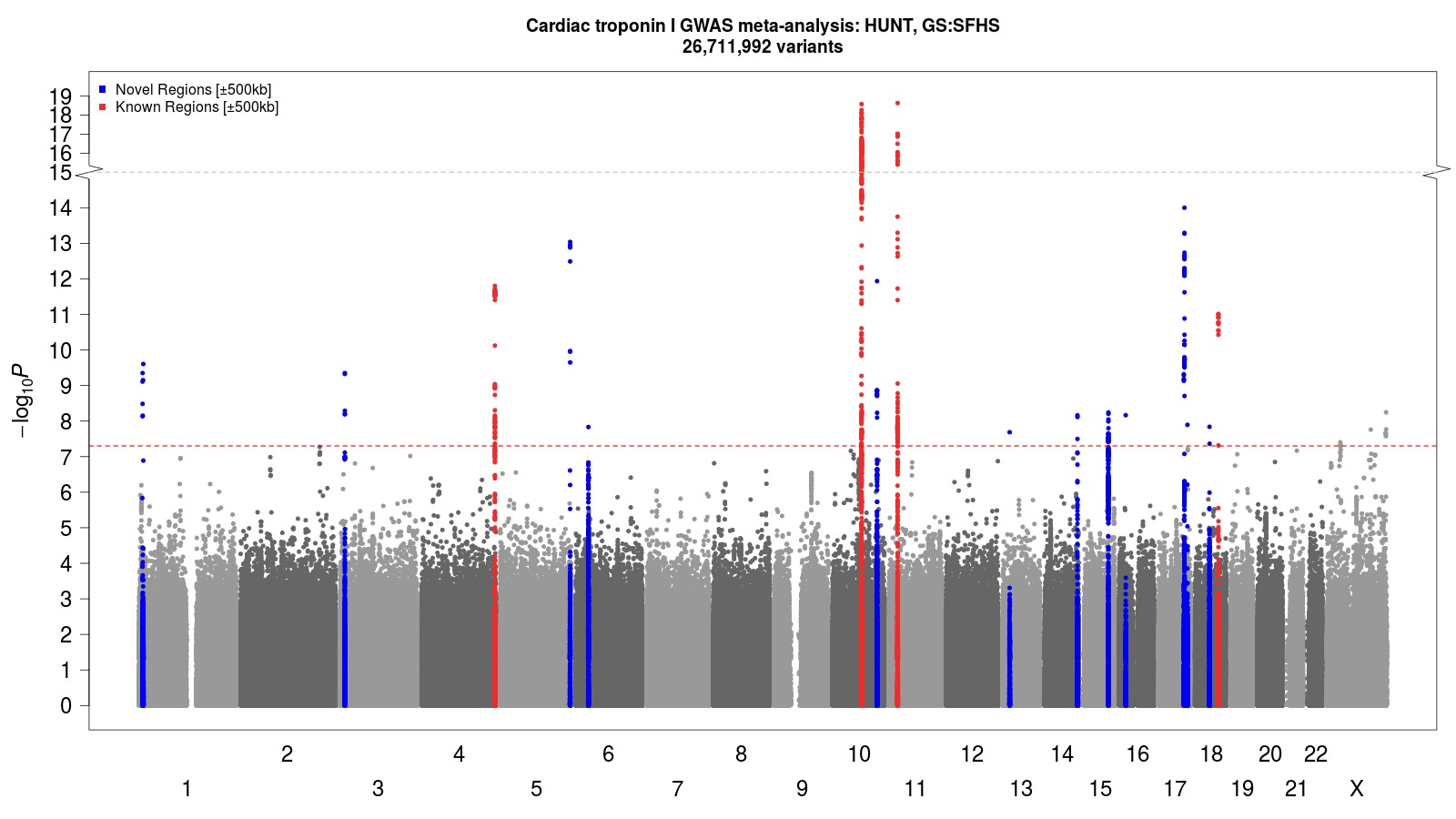


***Figure S1:*** *Manhattan plot: Genomic loci associated (p-value < 5×10^-8^ ) with cTnI concentration in HUNT + GS:SFHS GWAS meta-analysis.*

*Chromosomes are given on the x-axis and -log(p-value) for the genetic variants are given on the y-axis.*


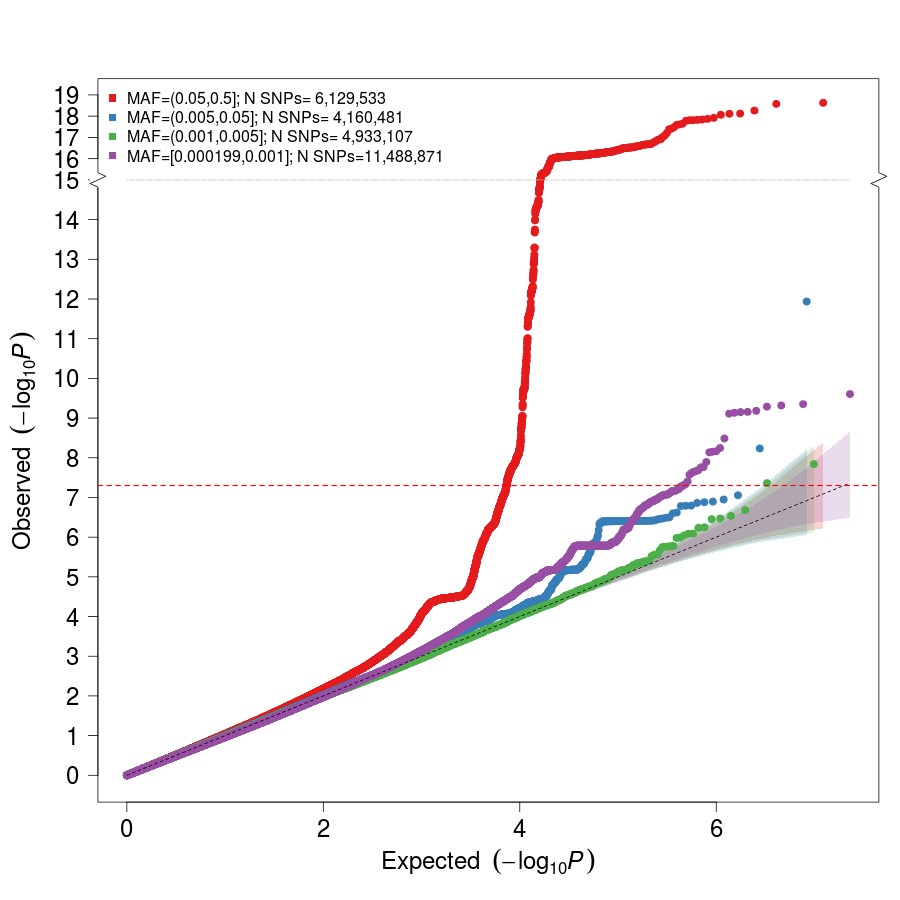


***Figure S2:*** *Quantile-quantile plot for HUNT + GS:SFHS GWAS meta-analysis summary statistics. The inflation factor* l *= 1.02 (based on single nucleotide variants with MAF > 0.01).*

# Author contributions

M.R.M analyzed the data and wrote the first draft of the manuscript. H.Røsjø contributed to the writing of the manuscript with knowledge on cardiac biology and cardiac troponin measurements. A.R, M.N.L, S.E.G, A.F.H, B.N.W, S.A.G.T, J.LF, H.Rasheed, L.F.T, W.Z, N.A., L.G.F, J.B.N and C.H contributed to analyses. B.M.B. and T.O. conceived and designed the study. All authors interpreted results and revised the paper.
